# Proline-Rich Homeodomain protein (PRH/HHEX) is a suppressor of breast tumour growth

**DOI:** 10.1038/oncsis.2017.42

**Published:** 2017-06-12

**Authors:** R M Kershaw, D Roberts, J Wragg, A M Shaaban, E Humphreys, J Halsall, L Price, R Bicknell, K Gaston, P-S Jayaraman

**Affiliations:** 1 Institute of Cancer and Genome Biology, College of Medical and Dental Sciences, University of Birmingham, Birmingham, UK; 2 School of Biochemistry, University of Bristol, Bristol, UK

## Abstract

Breast tumours progress from hyperplasia to ductal carcinoma *in situ* (DCIS) and invasive breast carcinoma (IBC). PRH/HHEX (proline-rich homeodomain/haematopoietically expressed homeobox) is a transcription factor that displays both tumour suppressor and oncogenic activity in different disease contexts; however, the role of PRH in breast cancer is poorly understood. Here we show that nuclear localization of the PRH protein is decreased in DCIS and IBC compared with normal breast. Our previous work has shown that PRH phosphorylation by protein kinase CK2 prevents PRH from binding to DNA and regulating the transcription of multiple genes encoding growth factors and growth factor receptors. Here we show that transcriptionally inactive phosphorylated PRH is elevated in DCIS and IBC compared with normal breast. To determine the consequences of PRH loss of function in breast cancer cells, we generated inducible PRH depletion in MCF-7 cells. We show that PRH depletion results in increased MCF-7 cell proliferation in part at least due to increased vascular endothelial growth factor signalling. Moreover, we demonstrate that PRH depletion increases the formation of breast cancer cells with cancer stem cell-like properties. Finally, and in keeping with these findings, we show that PRH overexpression inhibits the growth of mammary tumours in mice. Collectively, these data indicate that PRH plays a tumour suppressive role in the breast and they provide an explanation for the finding that low PRH mRNA levels are associated with a poor prognosis in breast cancer.

## Introduction

Ductal carcinoma *in situ* (DCIS) is a non-invasive breast carcinoma with increasing incidence. It comprises a proliferation of neoplastic epithelial cells within mammary ducts with or without lobular involvement. DCIS can progress over time to invasive breast carcinoma (IBC).^1^ Breast cancer formation and progression occurs through random changes in genes and gene expression, resulting in clonal expansion of those cells that have an advantageous phenotype. Tumour-initiating cells have stem cell-like properties and are also known as cancer stem cells (CSC). In current models of breast tumour progression, CSC are believed to be derived from transit-amplifying cell populations that exist within normal mammary stem cell differentiation. The transit-amplifying cells are more highly proliferative than true mammary stem cells, but they are still capable of self-renewal and differentiation along multiple lineages, (reviewed in Chaffer and Weinberg^2^ and Ye and Weinberg^3^). An important property of CSC is that they can produce differentiated progeny, that is, bulk cancer cells without self-renewal properties, and this differentiation is reversible so the bulk cancer cells can dedifferentiate back towards CSC.^4, 5^ Members of the Zeb, Twist, Slug and Sox9 transcription factor families are known to promote morphological changes known as epithelial to mesenchymal transition, whereby epithelial cells acquire a mesenchymal phenotype and become elongated and migratory. Although this alteration was initially believed to be associated with tumour progression towards invasion, it is now also linked with tumour initiation and progression as the same factors promote CSC formation (reviewed in Ye *et al.*^6^). Transcription factors from the Zeb family, for example, regulate the expression of the CSC marker CD44 and govern the propensity of breast cells from different lineages to become CSC.^7^

PRH/HHEX (proline-rich homeodomain/haematopoietically expressed homeobox) is a DNA-binding protein that regulates the development of multiple tissues in the embryo and tissue homoeostasis in the adult. PRH misregulation is associated with a variety of cancers and leukaemias (reviewed in Gaston *et al.*^8^and Soufi and Jayaraman^9^). Although PRH can function as an oncogene in some subtypes of leukaemia, it has been shown to possess tumour suppressor activity in acute myeloid leukaemia cells and in liver tumour cells (reviewed in Gaston *et al.*^8^). PRH regulates cell proliferation via multiple mechanisms. PRH directly regulates the transcription of several genes encoding growth factors, such as *VEGFA*, growth factor receptors, including *FLT1* (VegfR1) and *KDR* (VegfR2) and inhibits VEGF autocrine signalling.^10, 11^ It also regulates the transcription of genes encoding growth factor co-receptors, such as the TGFβ co-receptor Endoglin, to control cell proliferation and cell migration.^12^ The DNA-binding activity of PRH is inhibited following the phosphorylation of amino acids in the PRH homeodomain by protein kinase CK2, preventing the regulation of these genes.^11^ In addition, PRH interacts directly with a variety of transcription factors and translation factors involved in the control of cell proliferation, including c-Myc, eIF4E and PML, modulating their activity and/or their intracellular localization.^13, 14, 15, 16^

Decreased nuclear localization of PRH has been observed in invasive breast ductal and lobular carcinomas (IBC).^17^ Here we use immunohistochemistry (IHC) and observe decreased nuclear PRH in human breast tumours and alterations in phosphorylated PRH in tumours compared with normal mammary epithelial cells. We demonstrate that PRH regulates breast cell proliferation and that PRH overexpression inhibits mammary tumour growth in mice.

## Results

### PRH expression and phosphorylation is altered in primary breast tumours

**Figure 1:**
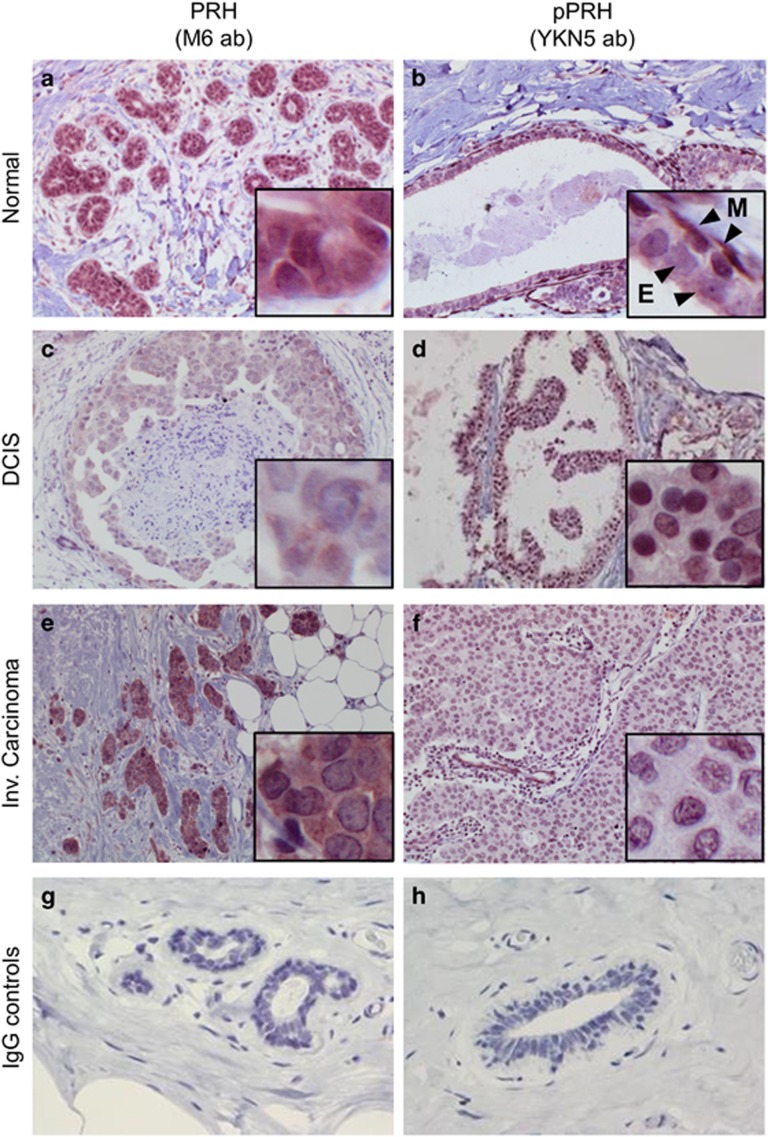
PRH and pPRH in normal breast, DCIS and invasive breast carcinoma. Representative images showing PRH and pPRH expression in normal breast (**a** and **b**: × 100 magnification), DCIS (**c** and **d**: × 200 magnification) and invasive carcinoma (**e** and **f**: × 200 magnification) determined using IHC. Total PRH was detected using the M6 monoclonal antibody at 1:2000 dilution (**a**, **c**, **e**). pPRH was detected using the YKN5 antibody at 1:3000 dilution (**b**, **d**, **f**) as described in the text. In **b** the inset shows the pPRH immunoreactivity of myoepithelial cells (M) and the weak pPRH immunoreactivity of luminal epithelial cells (E). (**g**, **h**) Show negative controls of normal tissue stained using mouse and rabbit IgG, respectively (× 200 magnification).

We examined PRH and pPRH expression in 14 normal breast sections, 7 DCIS and 13 IBC cases using IHC (Figure 1 and Summarized in Table 1). Figure 1 shows representative images in which either PRH or pPRH are stained red (NovaRed substrate) and cell nuclei are counterstained blue with haematoxylin. The tissue samples were assessed for cytoplasmic and nuclear PRH and pPRH staining across the whole slide and categorized into low percentage (0–10), intermediate percentage (11–70) or high percentage (71–100) of cells with positive staining. In addition, the intensity of staining was similarly categorized into very weak, weak, intermediate or strong (Table 1). The IHC analysis was performed by a specialist breast pathologist. In normal breast, PRH is present in both the nucleus and the cytoplasm of ductal epithelial cells (Figure 1a). In DCIS and IBC there is weak PRH staining in the nuclei in 7/7 and 10/12 cases, respectively (Figures 1c and e). Some invasive carcinomas also show strong or intermediate cytoplasmic PRH staining intensity, but this is variable and also present in some DCIS (2/7) and some apparently normal samples (2/13). Phosphorylated PRH staining is generally very weak in the cytoplasm and nuclei of normal luminal epithelial cells (Figure 1b). However, pPRH is strongly expressed in surrounding myoepithelial cells (inset, Figure 1b) and in some normal breast samples (3/12). Most DCIS cases show strong nuclear pPRH expression and the majority of nuclei are stained (Figure 1d). A statistical comparison of categorical data for staining intensity for normal breast, DCIS and IBC and a similar analysis of categorical data for area stained for normal breast, DCIS and IBC showed that there is a highly significant decrease in the percentage of nuclei stained for PRH (*P*=2 × 10^−3^) and decreased nuclear staining intensity (*P*=2 × 10^−4^) in tumours compared with normal breast tissue and a trend towards increased cytoplasmic staining. There is also a statistically highly significant increase in the percentage of nuclei stained for pPRH (*P*=7.14 × 10^−3^) and in the intensity of staining (*P*=4.76 × 10^−3^) in DCIS compared with normal tissue. Although a similar trend is observed in IBC compared with normal, this was not statistically significant. These data confirm that PRH is less nuclear in breast carcinomas and is similar to the data previously reported by Puppin *et al.*^17^ Moreover, these data show that transcriptionally inactive pPRH is dramatically increased in DCIS and also elevated in IBC.

### PRH regulates the proliferation of MCF-7 cells

**Figure 2:**
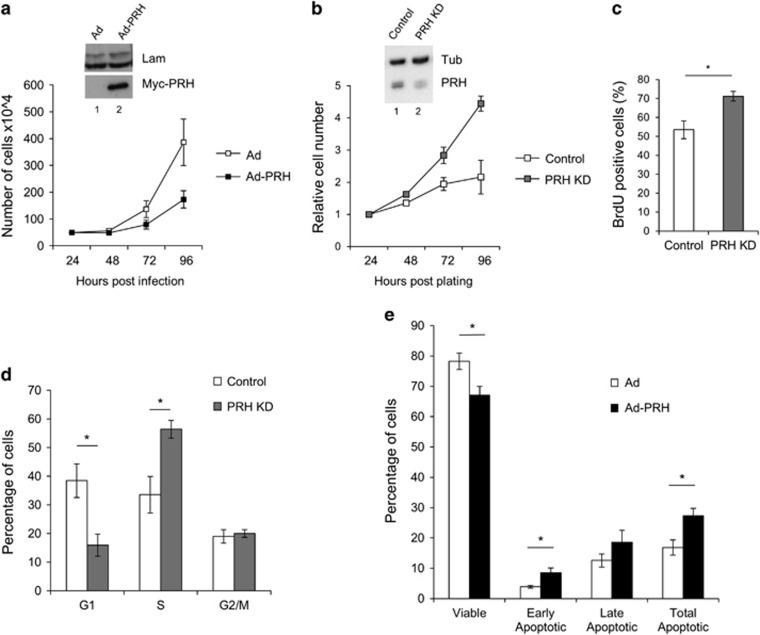
PRH regulates the proliferation of MCF-7 cells. (**a**) MCF-7 cells were infected with control adenovirus (empty symbols) or an adenovirus expressing Myc-tagged PRH (filled symbols) at an MOI of 50. The number of viable cells was determined using an MTT assay at the time points indicated post-infection. Mean and standard deviation (s.d.) from *n*=3 independent experiments each performed in triplicate. The inseted panel shows a western blot for Myc-PRH in the infected cells 24 h post-infection with Lamin A/C as a loading control. (**b**) MCF-7 control and PRH KD cell lines were induced with IPTG for 7 days. Cell number was then determined by MTT assay over 72 h. (mean and s.d., *n*=3 independent experiments). The inseted panel shows a western blot for PRH (M6 antibody) at day 7 post-induction and with Tubulin as a loading control. (**c**) Cell proliferation following PRH KD was determined by BrdU incorporation 7 days post-induction. The graph shows the percentage of BrdU-positive cells (mean and s.d., *n*=3, *Student’s *t*-test *P*<0.05). (**d**) PRH KD and control cells 7 days post-induction were dual stained with PI/AV (APC antibody) and analysed by flow cytometry. The graph shows the percent distribution of cells in each stage of the cell cycle (mean and s.d., *n*=3). (**e**) MCF-7 cells were incubated with control Ad or Ad-PRH for 4 days before being stained with 10 μg/μl propidium iodide and 5 μl of Annexin V-APC antibody (BD Biosciences - Europe, Oxford, UK). Cells were then analysed by flow cytometry to determine live cells (PI−, AV−) or apoptotic cells (PI−, AV+ or PI+, AV+) (*n*=3, two-tail homoscedastic *t*-test *P<0.05).

To examine the role of PRH in breast cells we overexpressed the protein in human breast adenocarcinoma MCF-7 cells using an adenoviral vector expressing Myc-tagged PRH and examined the effects on cell number in MTT cell viability assays. Infection with Ad-PRH resulted in a decrease in cell number with time compared with cells infected with empty adenovirus (Figure 2a). Western blotting confirmed expression of Myc-PRH in the Ad-PRH-infected cells (Figure 2a, inset). We have observed previously that transient PRH knockdown (KD) in MCF-7 cells increases cell number.^10^ To confirm this we made use of an isopropyl β-d-1-thiogalactopyranoside (IPTG)-inducible lentiviral vector expressing PRH shRNA to knockdown PRH expression. Three independent PRH KD cell lines or control cell lines (transduced using IPTG-inducible control shRNA expressing viruses) were generated from MCF-7 cells to ensure that the sites of viral integration differ in each cell line. PRH KD in IPTG-treated MCF-7 cells transduced with the inducible PRH shRNA vector was observed using western blotting (Figure 2b, inset). PRH KD in these independent cell lines resulted in increased cell number in MTT cell viability assays (Figure 2b) and increased cell proliferation as measured using a BrdU incorporation assay (Figure 2c). Moreover, propidium iodide staining of DNA content and flow cytometry showed that in the PRH KD cells there is a decrease in the percentage of cells in G1 and an increase in the percentage of cells in S-phase (Figure 2d). In contrast, PRH overexpression in MCF-7 cells resulted in a significant decrease in the number of viable cells and an increase in apoptosis (Figure 2e). These results clearly demonstrate that PRH negatively regulates the proliferation of MCF-7 breast cancer cells. PRH overexpression can also inhibit the proliferation of MDA-MB-231 breast cancer cells. In these cells PRH overexpression resulted in an increase in the percentage of cells in G1 (Supplementary Figure S1A) and a reduction in cell proliferation as measured by BrdU incorporation (Supplementary Figure S1B).

### PRH regulates the expression of proliferation-, angiogenesis- and CSC-related genes

**Figure 3:**
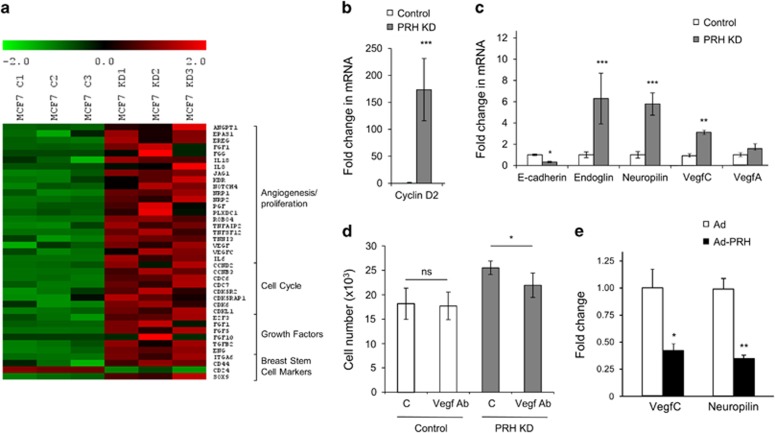
Altered gene expression in MCF-7 PRH KD cells. (**a**) mRNA expression data are presented as a matrix in which rows represent individual genes, and columns represent individual mRNA samples from three independent control (C1–C3) and PRH KD (KD1–KD3) cell lines. The relative level of gene expression is depicted according to the colour scale shown. From 4980 differentially expressed genes selected genes with known function in angiogenesis/cell proliferation, cell cycle, growth factors and breast stem cell markers are shown. (**b**) Relative expression of *CCN2* (Cyclin D2) was determined by RT–PCR normalized to *GAPDH* mRNA (*n*=3. Student’s *t*-test, ***P<0.001). (**c**) Relative expression of *CDH1* (E-Cadherin), *ENG* (Endoglin), *NRP1* (Neuropilin), *VEGFC* and *VEGFA* was determined as above (*n*=3, Student’s *t*-test, *P<0.05, **P<0.01, ***P<0.001). (**d**) Control cells and PRH KD MCF-7 cells were grown in the absence or presence of VEGF antibody. Cell number was determined using an MTT assay (*n*=3, Student’s *t*-test, *P<0.05). (**e**) The relative expression of *NRP1* (Neuropilin) and *VEGFC* in MCF-7 cells infected with empty Adenovirus (Ad) and Ad-PRH was determined by RT–PCR normalized to *GAPDH* mRNA (*n*=3, Student’s *t*-test, *P<0.05, **P<0.01, ***P<0.001).

To better understand which genes are regulated by PRH in breast cells, we performed mRNA microarray experiments (Figure 3a). In total, 2299 genes were significantly upregulated and 2681 genes downregulated following PRH KD (fold change >1.5 statistical analysis of microarray false discovery rate *P*<0.05). Interestingly, GO pathway analysis (GATHER) revealed that 165 genes associated with the term cell cycle (GO:000074), 55 genes associated with the term proliferation (GO:0042127) and 20 genes associated with the term angiogenesis (GO:0001525) were significantly upregulated in the PRH KD cells including *VEGFA* and *VEGFC*. Additionally genes known to encode CSC markers (*CD44*, *CD24* and *ITGA6*) were significantly altered. Selected genes are shown in a heat map (Figure 3a). To validate the microarray data quantitative RT–PCR was performed on several altered genes encompassing a wide range of fold change in expression values: *CCND2* (x179), *NRP1* (x3), *ENG* (x12), *VEGFA* (5 transcripts x1.8–x3.5) and *VEGFC* (x3.5). In each case the mRNAs were altered in accordance with the change observed in the microarray data (Figures 3b and c).

Since *VEGFA*, *VEGFC, NRP1* and *VEGFR2* are altered in the microarray data and upregulation of *KDR* (VegfR2) and *VEGFA* has been observed by us before in transient PRH KD experiments,^10^ we set out to determine whether inducible PRH KD also results in cells that have an altered autocrine response to VEGF signalling. We carried out MTT cell viability assays with MCF-7 control and MCF-7 PRH KD cells in the presence and absence of a VEGF antibody^18^ (Figure 3d). PRH KD cell numbers decreased upon addition of the VEGF antibody whereas control cell numbers were not significantly altered (Figure 3d). Since cell number is reduced by the VEGF antibody only in PRH KD cells, we conclude that increased VEGF autocrine signalling contributes to the increased proliferation of these PRH KD cells.

To determine whether any of the genes upregulated in the PRH KD cells are downregulated during PRH overexpression, MCF-7 cells were infected with Ad-PRH or an empty adenovirus as described above and RNA extracted for microarray experiments. The results of Myc-tagged PRH overexpression on selected genes are presented in Supplementary Figure S2. Expression of Myc-PRH downregulated 2633 genes and upregulated 1913 genes (fold change >1.5 statistical analysis of microarray false discovery rate *P*<0.05). GO analysis (GATHER) showed that 41 cell cycle (GO:0007049) and 8 angiogenesis genes (GO:00001525) are downregulated upon PRH overexpression including *VEGFC* (× 0.32) and *NRP1* (× 0.47). To validate the overexpression microarray the expression of *VEGFC* and *NRP1* was examined using quantitative PCR and shown to be downregulated in agreement with the microarray data (Figure 3e). *VEGFC* expression has recently been shown to be correlated with mammary tumour proliferation^19, 20^ and *VEGFC* falls within the subset of 414 genes that are inversely regulated, that is upregulated in PRH KD but downregulated by PRH overexpression. Changes in VEGFC may therefore contribute to the growth phenotype observed upon perturbation of PRH levels.

### PRH knockdown promotes the formation of CSC-like cells

**Figure 4:**
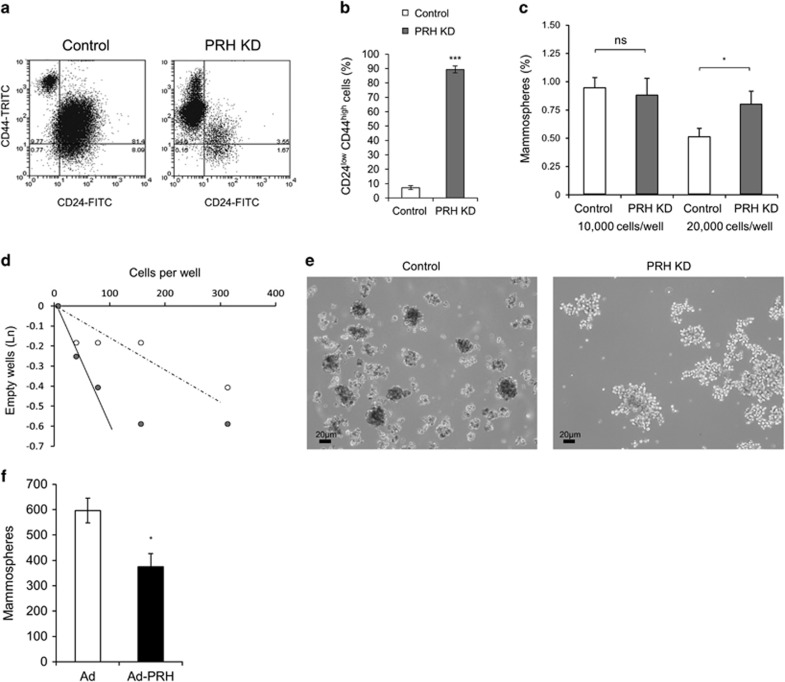
PRH levels influence mammosphere formation. MCF-7 control and PRH KD cell lines were induced with IPTG for 7 days then analysed by flow cytometry. (**a**) Flow cytometry analysis of the CSC markers CD24 and CD44. (**b**) Quantification of CD24^lo^CD44^hi^ cells (*n*=4, Student’s *t*-test, ***P<0.005). (**c**) Quantification of primary mammospheres formed by control and PRH KD MCF-7 cells (*n*=3, Student’s *t*-test, *P<0.05). (**d**) Limiting dilution analysis with secondary MCF-7 control and PRH KD mammospheres counted at day 14, (*n*=5). (**e**) Mammospheres and mammosphere-like particles were imaged under optical light (mag. × 10). (**f**) Quantification of primary mammospheres formed by control and PRH overexpressing MCF-7 cells (*n*=3, Student’s *t*-test, *P<0.05).

Our microarray studies showed that PRH KD cells have altered expression of CD44 and CD24 genes and also of breast CSC-related genes such as Sox9 and ITGA6 (Figure 3a). Flow cytometry analysis showed that there is a dramatic increase in the CD44^hi^/CD24^lo^ population in PRH KD cell lines compared with controls (Figures 4a and b) corresponding to the changes in gene expression in the microarray (Figure 3a). These data suggest that the PRH KD population contains an increased number of cells with a CSC-like gene expression pattern. To further examine the formation of these CSC-like cells, we performed mammosphere formation assays under non-adherent conditions. Significantly more mammospheres (>50μm size particles) were produced by MCF-7 PRH KD cells compared with controls when seeded at 20 000 cells/well but this was not observed at a lower cell density, possibly due to altered autocrine cell signalling at high cell densities (Figure 4c). Limited dilution assays were performed to examine secondary mammosphere formation^21^ and these assays confirm that the PRH-depleted cells are better able to form mammospheres as judged by the steep initial gradient for PRH KD cells compared with control cells (Figure 4d). However, at higher cell numbers there is departure from a linear relationship for the PRH KD cells suggestive of altered cell adhesion. Interestingly the PRH KD mammospheres are large extended structures that are very different in appearance from the compact spherical structures produced by control cells (Figure 4e). We conclude that PRH depletion results in increased formation of CSC-like cells and/or CSC progeny (amplifying progenitors) that retain the stem cell marker phenotype (CD44^hi^/CD24^lo^) and that these cells contribute to the large mammosphere-like particles.

To determine whether overexpression of PRH inhibits formation of the CSC-like cells, we infected MCF-7 cells with Ad-PRH or control virus and determined the number of primary mammospheres formed 7 days post-infection. As expected, overexpression of PRH decreased the number of mammospheres formed (Figure 4f). However, PRH overexpression did not alter expression of CD44 and CD24 marker proteins in cells prior to mammosphere formation at 48 h post-infection as measured using flow cytometry (Supplementary Figure S3). In summary, downregulation of PRH leads to increased expression of CD44 and decreased expression of CD24 and increased mammosphere formation in limiting dilution analysis. Although overexpression of PRH leads to formation of fewer mammospheres, CD44 and CD24 are not altered under these conditions. This suggests that the reduction of mammosphere number following PRH overexpression may be indirect through an inhibitory effect on cell proliferation rather than a direct effect on CSC genes.

### PRH can act as a breast tumour suppressor

**Figure 5:**
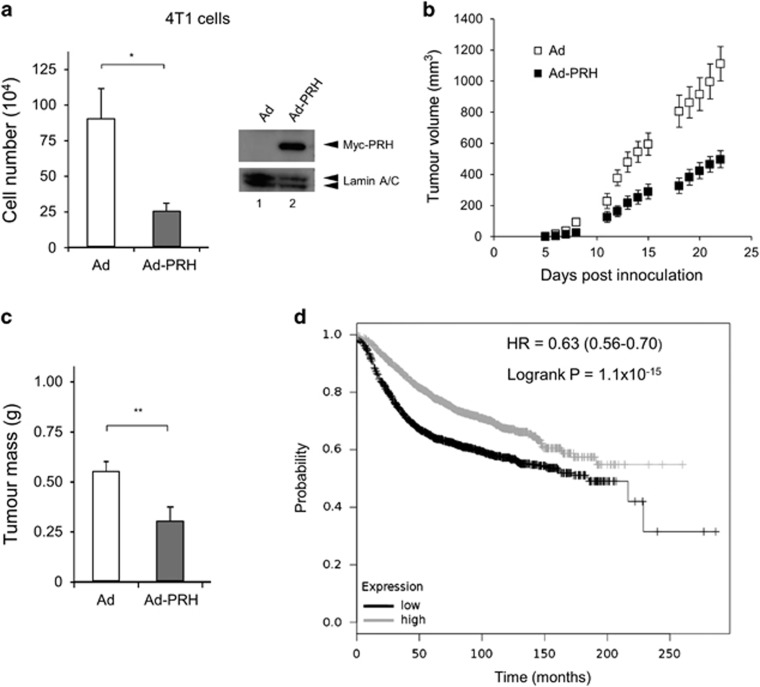
PRH overexpression decreases tumour growth. (**a**) Left—number of 4T1L cells 6 days after infection with Ad-PRH. Cells infected with Ad-PRH or empty adenovirus (Ad) were plated at equal numbers at 24 h and viable cells were counted at day 6 post-infection (*n*=3, Student’s *t*-test *P<0.05). Right—Western blot with 4T1L cells infected with Ad or Ad-PRH for 48 h. Lamin A/C as a loading control. (**b**) Tumour volumes as calculated by caliper measurements (width × height × depth) for each time point following injection of Ad-PRH or Ad-infected 4T1L cells into BALB/C mice (*n*=10, Student’s *t*-test, *P*<0.001 at day 22, combined data from two independent experiments). (**c**) Tumours were excised from the mice in (**b**) and weighed (*n*=10, **P<0.01). (**d**) Kaplan–Meier survival plot for PRH expression and probability of survival: black=low expression, grey=high expression. Logrank test *P*=1.1−10^−15^.

To directly examine the importance of PRH activity in tumour growth we overexpressed PRH in mouse mammary tumour cells. We used murine 4T1 mammary tumour cells for these experiments because when they are introduced orthotopically into syngeneic mice they are capable of rapid tumour initiation. Thus 4T1 cells expressing luciferase (4T1-12B cells) are useful for non-invasive imaging of *in vivo* tumour growth.^22^ We modified the 4T1 cell line to express high levels of luciferase using a lentiviral-luciferase expression vector (creating 4T1L cells). As expected, PRH overexpression in 4T1L cells reduced cell number *in vitro* (Figure 5a). Western blotting shows that the Myc-tagged PRH protein is highly expressed in the 4T1L cells 48 h post-infection (Figure 5a). Orthotopic injection of 4T1L cells into the mammary fat pad of syngeneic BALB/C mice resulted in tumours that formed within 8–12 days (Figure 5b). However 4T1L cells overexpressing PRH produced significantly smaller tumours than control cells (Figures 5b and 5c). These data suggest that PRH has a tumour suppressor role in breast epithelial cells.

### Low PRH mRNA expression correlates with decreased breast cancer survival

Since PRH expression is altered in breast cancer cells and PRH depletion in MCF-7 cells results in increased cell proliferation and increased formation CSC-like cells, we examined public databases for evidence linking PRH expression to breast tumour formation and breast tumour progression. The Kaplan–Meier plotter database contains survival information and mRNA expression data for breast tumour patients.^23^ Figure 5d shows a Kaplan–Meier plot generated from these data. Low expression of PRH mRNA in breast tumour patients across all breast tumour subtypes correlates with a poorer relapse-free survival compared with patients expressing higher levels of PRH (*P*>10^−14^). A similar outcome was observed using the Gene Expression-Based Outcome for Breast Cancer Online (GOBO) database^24^ (Supplementary Figure S4). Consistent with these data interrogation of the MethHC database that reports on DNA methylation and mRNA expression shows that the *HHEX* (PRH) gene is hypermethylated in breast cancer (Supplementary Figure S5A) and that increased methylation is associated with lower mRNA expression (Supplementary Figure S5B). Thus, *HHEX* gene expression appears to be downregulated in breast cancer cells and this is associated with a poor outcome.

## Discussion

The PRH protein is known to regulate the proliferation of multiple cell types, including haematopoietic lineages, vascular cells and liver hepatocytes. Decreased nuclear localization of PRH has been observed in thyroid tumours and in subtypes of acute myeloid leukaemia and in both ductular and lobular IBCs.^16, 17, 25^ In liver cancer cells, PRH overexpression downregulates tumour growth in mouse xenograft models and PRH antagonizes c-Myc activity.^13, 26^ In well-differentiated liver tumours, there is little nuclear PRH expression and strong cytoplasmic expression, whereas in poorly differentiated tumours there is lower cytoplasmic expression of PRH. However, not all tumours follow this trend in that a fraction of poorly differentiated tumours show high nuclear PRH expression.^26^ Thus, it is not yet clear whether a reduction in PRH expression accompanies a loss in differentiation in this tumour type. In prostate cells PRH plays a tumour suppressor role. We have demonstrated that decreasing active PRH in immortalized prostate epithelial cells promotes both cell proliferation and cell migration.^27^ Moreover, we have demonstrated that pPRH is elevated in prostate cancer cell lines, in benign prostatic hyperplasia and prostatic adenoarcinoma compared with normal controls.^27^ Thus, loss of nuclear localization, decreased PRH expression and elevated phosphorylation of PRH are associated with tumourigenesis.

Examination of PRH expression and localization in a cohort of primary breast tissues samples using IHC shows that PRH protein is present in normal breast tissues in nuclear and cytoplasmic compartments but is less strongly nuclear and more cytoplasmic in DCIS and IBC. These results are broadly in agreement with the observations of Damante and co-workers;^17^ however, they did not measure PRH levels in normal breast tissues or in *in situ* carcinomas and used different antibodies so direct comparisons are difficult. Phosphorylated PRH is present only at low levels in the cytoplasm of normal primary breast epithelial cells but pPRH becomes greatly elevated in the nuclei of luminal epithelial cells in DCIS and elevated to a lesser extent in IBC. One possible explanation for the increased pPRH observed is that increased inactivation of PRH occurs when these cells proliferate, whereas when they become invasive, they may show increased phosphorylation in conjunction with decreased expression and/or altered subcellular localization. Larger sample sizes are required to allow an investigation of PRH and PRH phosphorylation status with tumour progression.

In agreement with the hypothesis that PRH is a growth control protein in breast cells that is inactivated during tumour progression, we have shown that PRH overexpression inhibits mammary tumour growth in a mouse model of breast cancer. In previous studies we showed that PRH overexpression in MDA-MB-231 breast cancer cells also inhibits cell migration and significantly reduces the ability of these cells to invade Matrigel *in vitro.*^12^ We have shown here that inducible knockdown of PRH in MCF-7 breast tumour cells results in additional tumorigenic properties including increased cell proliferation, due in part to increased expression of cell cycle genes, to increased autocrine VEGF signalling through VEGFA and most likely VEGFC, and increased mammosphere forming frequency. Indeed VEGFC is known to increase CSC formation through autocrine signalling via NRP2 in Claudin-low breast cells.^28^ In culture CSC are in a dynamic equilibrium between CSC and non-CSC daughter cells through autoregulatory inflammatory IL6- and IL-8-dependent feedback loops.^4, 29^ The MCF-7 PRH KD cells have increased expression of both IL-8 (× 33) and IL6 (× 2.7) (microarray data). Thus, autocrine signalling through these cytokines and increased autocrine survival signalling through growth factors such as VEGFA and VEGFC are likely to contribute cumulatively to the stable increase in cells with a CSC marker phenotype and to altered mammosphere formation.

Together these data support the notion that PRH is a breast tumour suppressor and that its nuclear activity may be compromised in breast tumour cells by increased PRH phosphorylation and/or decreased PRH mRNA expression and altered subcellular localization. However, it is important to point out that PRH can also have oncogenic activities and like many other factors involved in tumorigenesis, PRH may play a tumour suppressor role in early breast cancer development and an oncogenic role at later time points. This is perhaps likely since PRH regulates the expression of many growth factors and growth factors and the impact of changes in PRH activity are likely to vary depending on both the extracellular environment and the intracellular signalling milieu. Nevertheless, given the correlation observed between low PRH mRNA levels and poor breast tumour survival as well as the changes in PRH localization and activity in DCIS and IBC, we propose that monitoring PRH protein levels or activity could be particularly important for assessing breast tumour prognosis. In addition, since PRH is known to be important in multiple cell types, this work has important implications for other types of cancer.

## Materials and methods

### Expression vectors and reporters

pMUG1-Myc-PRH expresses human PRH tagged with the Myc9E10 epitope.^30^ Lentiviral constructs expressing IPTG-inducible PRH shRNA or control shRNA (Sigma, St Louis, MO, USA) are described in Kershaw *et al.*^12^ Adenoviral construct expressing Myc-PRH is described in Soufi *et al.*^31^

### Cell culture, PRH knockdown and transient transfection

Culture and transfection of MDA-MB-231 and MCF-7; early passage cells obtained from ATCC (Manassas, VA, USA).^12^ PRH KD and lentiviral infection and induction of IPTG-inducible PRH shRNA in MCF-7 cells has been described previously.^12^ Briefly, MCF-7 cells were transduced using a control lentivirus that activates RNA-induced silencing complex and the RNA interference pathway but does not target any known gene or a lentivirus expressing PRH shRNA. shRNA expression was then induced using IPTG for 7 days. In both cell lines multiple independent KD and control cell lines were generated using the same vectors and MCF-7 control and KD cells were used following 7 days of IPTG induction with 1 mM IPTG.

### Quantitative reverse transcriptase-mediated PCR (RT–qPCR)

RNA was purified 7 days post-IPTG induction.^10, 32^ Quantitative PCR was performed in triplicate with gene of interest and glyceraldehyde-3-phosphate dehydrogenase primers (Supplementary Table S1). Data analysis: Rotorgene software, Qiagen, (Rotor-gene Q 5plex HRM), glyceraldehyde-3-phosphate dehydrogenase mRNA was used as an internal control and fold change was analysed by the efficiency adjusted quantitative PCR method.

### Western blotting and immunostaining

#### Westerns

Whole-cell extracts were prepared as described^33^ and 20 μg total protein was loaded. PRH antibodies have been described previously.^30, 32^ Lamin A/C and Tubulin were used as loading controls; these and secondary antibodies were from Santa-Cruz Biotechnology, Inc. (Dallas, TX, USA). Densitometry was performed using ImageJ software (NIH, Bethesda, MD, USA).

### Immunohistochemistry

Immunohistochemistry was performed on formalin-fixed paraffin-embedded human breast tissues (normal, DCIS, carcinoma). Tissues were ethically obtained from the Queen Elizabeth Hospital and University Birmingham pathology archive through the Human Biobank Repository Centre. The tissues were stained with the Vector ImmPress Excel anti-mouse Ig or anti-rabbit Ig peroxidase kits (MP-7602 or MP-7601, respectively, Vector Labs, Burlingame, CA, USA) and visualized with the ImmPact NovaRed peroxidase substrate (Vector Labs) according to the manufacturer’s instructions. Staining was performed was follows: tissues were dewaxed in xylene (PFM Medical, Poynton, Cheshire, UK) and then rehydrated in graded alcohols (VWR, Lutterworth, Leicestershire, UK) and then distilled water. Antigen retrieval was performed with high pH buffer (Vector Labs) and the sections were incubated in Bloxall solution (Vector Labs) for 10 min at 20 °C to block endogenous peroxidase activity and then in 2.5% horse serum (Vector Labs) for 20 min at 20 °C. The primary antibodies (monoclonal mouse anti-human PRH (M6) 1:2000 or polyclonal rabbit anti-human phosphoPRH (YKN5) 1:3000) diluted in 2.5% horse serum were added to the sections and incubated overnight at 4 °C. The sections were then washed in Tris-buffered saline pH 7.2 for 5 min and incubated with amplifier antibody for 15 min at 20 °C. After washing in Tris-buffered saline pH 7.2 for 5 min the sections were incubated with the ImmPress Excel tertiary antibody for 30 min at 20 °C. The sections were washed in Tris-buffered saline pH 7.2 for 5 min and the NovaRed peroxidase substrate was added for 5 min. The reaction was stopped by washing in distilled water for 5 min and the sections counterstained using Mayers haematoxylin (PFM Medical) for 30 s. The sections were mounted using DPX (Cell Path, Newtown, Powys, UK) and left to dry before being visualized on an Olympus BX53 microscope (Olympus, Southend-on-Sea, Essex SS2 5QH, UK) with an Olympus SC100 Camera. IHC data were analysed by categorizing PRH or pPRH expression staining intensity as very weak, weak, intermediate and strong. Number of PRH or pPRH nuclei stained in relation to total cell number was categorized as low (0–10%), intermediate (11–70%) and high (71–100%). Statistical analysis of IHC intensity and percentage staining was performed using Kruskal–Wallis tests for categorical data of normal vs DCIS vs pooled carcinoma. For any test with *P*<0.05 *post hoc* Wilcoxon–Mann–Whitney tests were performed with Bonferroni corrections of Mann–Whitney *P*-values for multiple comparisons. The corrected *P*-values are reported.

### Microarrays

RNA from three independent control (C1–C3) and PRH KD (KD1–KD3) MCF-7 cell lines, extracted 7 days post-IPTG induction using a RNeasy mini kit (Qiagen, Manchester, UK), was used for double-stranded cDNA synthesis (Roche Products Limited (Pharmaceuticals), Welwyn Garden City, UK). cDNA was labelled with Cy3 (NimbleGen One-Color DNA labelling kit) then hybridized to a Roche NimbleGen 12 × 135 K gene expression microarray. The array was washed and scanned using the MS 200 Microarray Scanner. Data were extracted and normalized by robust multi-array analysis using DEVA software (v1.2.1). Genes showing significantly altered expression were identified by statistical analysis of microarray analysis using MeV software (v4.9). Gene lists were generated for significantly different expression levels using fold change greater than 1.5 and false discovery rate-adjusted *P*-values of <0.05. For overexpression microarray experiments RNA from cells infected in three independent experiments with empty Adenovirus or Adenoviral-PRH (moi 50) was extracted 48 h after infection. RNA production, cDNA labelling and hybridization, washing and SAM microarray analysis were performed exactly as described for the KD cells.

### Cell counting and proliferation assays

Cell counting and MTT assays as described in Noy *et al.*^10^ Cells were incubated with MTT for 2 h, solubilized with dimethyl sulfoxide and optical density was measured at 540 nm. For BrdU staining cells (5 × 10^5^) adhered to coverslips were incubated with 10 μm BrdU (Sigma) for 6 h and then fixed with 4% w/v formaldehyde. Endogenous peroxidase activity was blocked with 3% v/v H_2_O_2_ (Sigma) and then DNA denatured using 2 m HCl (Sigma). Cells were incubated with murine anti-BrdU antibody (Sigma) (1:500 in 1% w/v bovine serum albumin+10% v/v horse serum) overnight, followed by biotinylated horse anti-mouse IgG (Vector Laboratories) for 30 min and finally with Extravadin-peroxidase (Sigma) for 30 min. Cells were stained with 3,3′-diaminobenzidine solution (Sigma) and then counted.

### Tumoursphere assays

For mammosphere formation cells (1 × 10^4^ or 2 × 10^4^) were plated in poly-HEMA (Sigma)-coated six-well plates containing Mammocult medium plus proliferation supplements (Stem Cell Technologies, UK Ltd., Waterbeach, Cambridge, UK) and cultured at 37 °C in 5% CO_2_. Seven days later primary mammospheres larger than 50 μm were counted on a graticule. Mammospheres were trypsinized to a single-cell suspension through a 25G needle and 2 × 10^4^ cells were re-seeded. Secondary mammospheres were counted seven days later. Limited dilution analysis was performed as outlined in Rota *et al.*^21^

### Flow cytometry

Cells resuspended in 100 μl phosphate-buffered saline were stained for 15 min with CD24-FITC and CD44-TRITC or CD133-APC (BD Biosciences, G44-26) and then analysed using a FACS Analyser Cyan ADP (Beckman Coulter, Brea, CA, USA).

### 4T1L syngeneic tumour model

4T1 cells were infected with a firefly luciferase and puromycin resistance-containing retrovirus MSCV-Luc.^34^ Infected cells were incubated with puromycin (8 μg/ml) to select for cells with high puromycin resistance and high luciferase expression. 4T1L cells were infected with Adenoviral-PRH or empty Adenovirus at MOI 50 for 24 h. Cells were washed 4 × with phosphate-buffered saline, trypsinized, washed and resuspended in Opti-mem (Invitrogen, Life Technologies Ltd, Paisley, UK) at 1.25 × 10^6^ cells/ml and kept on ice for 20–30 min before injection. Aliquots 200 μl/2.5 × 10^5^ of the cells were injected into the no. 3 fat pad of 6- to 8-week-old female BALB/C mice. Experiments with animals were carried out in accordance with animal care guidelines (Birmingham University) and with home office requirements (Licence number, PPL 40/3339).

## Figures and Tables

**Table 1:**
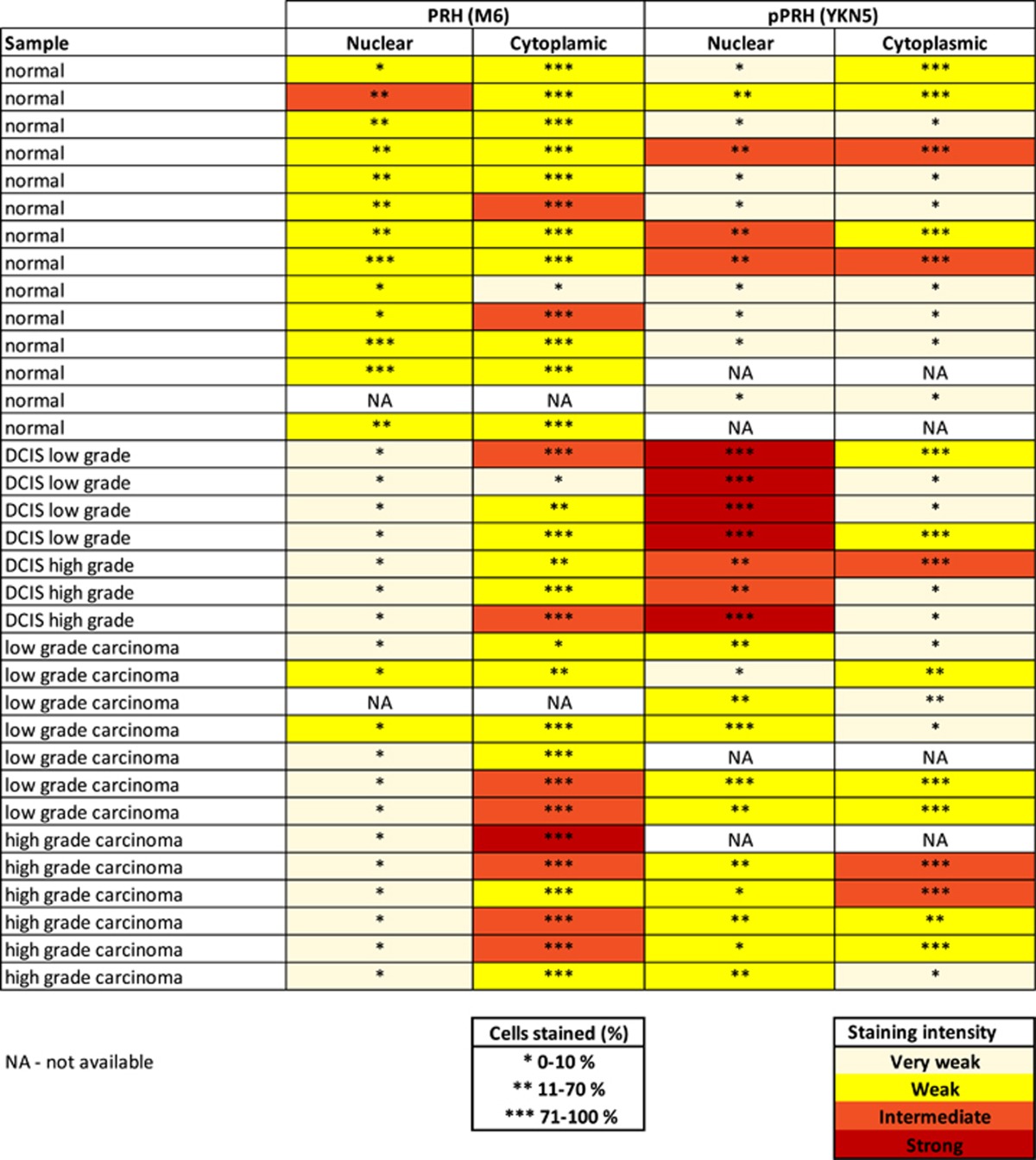
A summary of the immunohistochemistry data for pPRH and PRH

## References

[bib1] Espina V, Liotta LA. What is the malignant nature of human ductal carcinoma *in situ*? Nat Rev Cancer 2011; 11: 68–75. 21150936 10.1038/nrc2950 PMC3756606

[bib2] Chaffer CL, Weinberg RA. How does multistep tumorigenesis really proceed? Cancer Discov 2015; 5: 22–24. 25583800 10.1158/2159-8290.CD-14-0788 PMC4295623

[bib3] Ye X, Weinberg RA. Epithelial-mesenchymal plasticity: a central regulator of cancer progression. Trends Cell Biol 2015; 25: 675–686. 26437589 10.1016/j.tcb.2015.07.012 PMC4628843

[bib4] Iliopoulos D, Hirsch HA, Wang G, Struhl K. Inducible formation of breast cancer stem cells and their dynamic equilibrium with non-stem cancer cells via IL6 secretion. Proc Natl Acad Sci USA 2011; 108: 1397–1402. 21220315 10.1073/pnas.1018898108 PMC3029760

[bib5] Chaffer CL, Brueckmann I, Scheel C, Kaestli AJ, Wiggins PA, Rodrigues LO et al. Normal and neoplastic nonstem cells can spontaneously convert to a stem-like state. Proc Natl Acad Sci USA 2011; 108: 7950–7955. 21498687 10.1073/pnas.1102454108 PMC3093533

[bib6] Ye X, Tam WL, Shibue T, Kaygusuz Y, Reinhardt F, Ng Eaton E et al. Distinct EMT programs control normal mammary stem cells and tumour-initiating cells. Nature 2015; 525: 256–260. 26331542 10.1038/nature14897 PMC4764075

[bib7] Chaffer CL, Marjanovic ND, Lee T, Bell G, Kleer CG, Reinhardt F et al. Poised chromatin at the ZEB1 promoter enables breast cancer cell plasticity and enhances tumorigenicity. Cell 2013; 154: 61–74. 23827675 10.1016/j.cell.2013.06.005 PMC4015106

[bib8] Gaston K, Tsitsilianos MA, Wadey K, Jayaraman PS. Misregulation of the proline rich homeodomain (PRH/HHEX) protein in cancer cells and its consequences for tumour growth and invasion. Cell Biosci 2016; 6: 12. 26877867 10.1186/s13578-016-0077-7 PMC4752775

[bib9] Soufi A, Jayaraman PS. PRH/Hex: an oligomeric transcription factor and multifunctional regulator of cell fate. Biochem J 2008; 412: 399–413. 18498250 10.1042/BJ20080035 PMC2570084

[bib10] Noy P, Williams H, Sawasdichai A, Gaston K, Jayaraman PS. PRH/Hhex controls cell survival through coordinate transcriptional regulation of vascular endothelial growth factor signaling. Mol Cell Biol 2010; 30: 2120–2134. 20176809 10.1128/MCB.01511-09 PMC2863580

[bib11] Noy P, Sawasdichai A, Jayaraman PS, Gaston K. Protein kinase CK2 inactivates PRH/Hhex using multiple mechanisms to de-repress VEGF-signalling genes and promote cell survival. Nucleic Acids Res 2012; 40: 9008–9020. 22844093 10.1093/nar/gks687 PMC3467080

[bib12] Kershaw RM, Siddiqui YH, Roberts D, Jayaraman PS, Gaston K. PRH/HHex inhibits the migration of breast and prostate epithelial cells through direct transcriptional regulation of Endoglin. Oncogene 2014; 33: 5592–5600. 24240683 10.1038/onc.2013.496

[bib13] Marfil V, Blazquez M, Serrano F, Castell JV, Bort R. Growth-promoting and tumourigenic activity of c-Myc is suppressed by Hhex. Oncogene 2015; 34: 3011–3022. 25220416 10.1038/onc.2014.240

[bib14] Topcu Z, Mack DL, Hromas RA, Borden KL. The promyelocytic leukemia protein PML interacts with the proline-rich homeodomain protein PRH: a RING may link hematopoiesis and growth control. Oncogene 1999; 18: 7091–7100. 10597310 10.1038/sj.onc.1203201

[bib15] Topisirovic I, Culjkovic B, Cohen N, Perez JM, Skrabanek L, Borden KL. The proline-rich homeodomain protein, PRH, is a tissue-specific inhibitor of eIF4E-dependent cyclin D1 mRNA transport and growth. EMBO J 2003; 22: 689–703. 12554669 10.1093/emboj/cdg069 PMC140753

[bib16] Topisirovic I, Guzman ML, McConnell MJ, Licht JD, Culjkovic B, Neering SJ et al. Aberrant eukaryotic translation initiation factor 4E-dependent mRNA transport impedes hematopoietic differentiation and contributes to leukemogenesis. Mol Cell Biol 2003; 23: 8992–9002. 14645512 10.1128/MCB.23.24.8992-9002.2003 PMC309660

[bib17] Puppin C, Puglisi F, Pellizzari L, Manfioletti G, Pestrin M, Pandolfi M et al. HEX expression and localization in normal mammary gland and breast carcinoma. BMC Cancer 2006; 6: 192. 16854221 10.1186/1471-2407-6-192 PMC1550255

[bib18] Fraser HM, Dickson SE, Lunn SF, Wulff C, Morris KD, Carroll VA et al. Suppression of luteal angiogenesis in the primate after neutralization of vascular endothelial growth factor. Endocrinology 2000; 141: 995–1000. 10698175 10.1210/endo.141.3.7369

[bib19] Varney ML, Singh RK. VEGF-C-VEGFR3/Flt4 axis regulates mammary tumor growth and metastasis in an autocrine manner. Am J Cancer Res 2015; 5: 616–628. 25973301 PMC4396036

[bib20] Liu YC, Ma WH, Ge YL, Xue ML, Zhang Z, Zhang JY et al. RNAi-mediated gene silencing of vascular endothelial growth factor C suppresses growth and induces apoptosis in mouse breast cancer *in vitro* and *in vivo*. Oncol Lett 2016; 12: 3896–3904. 27895746 10.3892/ol.2016.5158 PMC5104198

[bib21] Rota LM, Lazzarino DA, Ziegler AN, LeRoith D, Wood TL. Determining mammosphere-forming potential: application of the limiting dilution analysis. J Mammary Gland Biol Neoplasia 2012; 17: 119–123. 22678420 10.1007/s10911-012-9258-0 PMC3428520

[bib22] Miller FR. Tumor subpopulation interactions in metastasis. Invasion Metastasis 1983; 3: 234–242. 6677628

[bib23] Gyorffy B, Lanczky A, Eklund AC, Denkert C, Budczies J, Li Q et al. An online survival analysis tool to rapidly assess the effect of 22277 genes on breast cancer prognosis using microarray data of 1809 patients. Breast Cancer Res Treat 2010; 123: 725–731. 20020197 10.1007/s10549-009-0674-9

[bib24] Ringner M, Fredlund E, Hakkinen J, Borg A, Staaf J. GOBO: gene expression-based outcome for breast cancer online. PLoS ONE 2011; 6: e17911. 21445301 10.1371/journal.pone.0017911 PMC3061871

[bib25] D'Elia AV, Tell G, Russo D, Arturi F, Puglisi F, Manfioletti G et al. Expression and localization of the homeodomain-containing protein HEX in human thyroid tumors. J Clin Endocrinol Metab 2002; 87: 1376–1383. 11889211 10.1210/jcem.87.3.8344

[bib26] Su J, You P, Zhao JP, Zhang SL, Song SH, Fu ZR et al. A potential role for the homeoprotein Hhex in hepatocellular carcinoma progression. Med Oncol 2012; 29: 1059–1067. 21656028 10.1007/s12032-011-9989-6

[bib27] Siddiqui YH, Kershaw RM, Humphreys EH, Assis Junior EM, Chaudhri S, Jayaraman PS et al. CK2 abrogates the inhibitory effects of PRH/HHEX on prostate cancer cell migration and invasion and acts through PRH to control cell proliferation. Oncogenesis 2017; 6: e293. 28134934 10.1038/oncsis.2016.82 PMC5294245

[bib28] Wang CA, Harrell JC, Iwanaga R, Jedlicka P, Ford HL. Vascular endothelial growth factor C promotes breast cancer progression via a novel antioxidant mechanism that involves regulation of superoxide dismutase 3. Breast Cancer Res 2014; 16: 462. 25358638 10.1186/s13058-014-0462-2 PMC4303136

[bib29] Singh JK, Simoes BM, Howell SJ, Farnie G, Clarke RB. Recent advances reveal IL-8 signaling as a potential key to targeting breast cancer stem cells. Breast Cancer Res 2013; 15: 210. 24041156 10.1186/bcr3436 PMC3978717

[bib30] Swingler TE, Bess KL, Yao J, Stifani S, Jayaraman PS. The proline-rich homeodomain protein recruits members of the Groucho/Transducin-like enhancer of split protein family to co-repress transcription in hematopoietic cells. J Biol Chem 2004; 279: 34938–34947. 15187083 10.1074/jbc.M404488200

[bib31] Soufi A, Smith C, Clarke AR, Gaston K, Jayaraman PS. Oligomerisation of the developmental regulator proline rich homeodomain (PRH/Hex) is mediated by a novel proline-rich dimerisation domain. J Mol Biol 2006; 358: 943–962. 16540119 10.1016/j.jmb.2006.02.020

[bib32] Soufi A, Noy P, Buckle M, Sawasdichai A, Gaston K, Jayaraman PS. CK2 phosphorylation of the PRH/Hex homeodomain functions as a reversible switch for DNA binding. Nucleic Acids Res 2009; 37: 3288–3300. 19324893 10.1093/nar/gkp197 PMC2691835

[bib33] Desjobert C, Noy P, Swingler T, Williams H, Gaston K, Jayaraman PS. The PRH/Hex repressor protein causes nuclear retention of Groucho/TLE co-repressors. Biochem J 2009; 417: 121–132. 18713067 10.1042/BJ20080872 PMC2605961

[bib34] Fang L, Lee VC, Cha E, Zhang H, Hwang ST. CCR7 regulates B16 murine melanoma cell tumorigenesis in skin. J Leukoc Biol 2008; 84: 965–972. 18519742 10.1189/jlb.1107776 PMC2538602

