# Supplementary figures and images for: Proline-Rich Homeodomain protein (PRH/HHEX) is a suppressor of breast tumour growth
Source: Oncogenesis. 2017 Jun 12;6(6):e346–. doi: 10.1038/oncsis.2017.42 (PMC5519192; doi:10.1038/oncsis.2017.42)

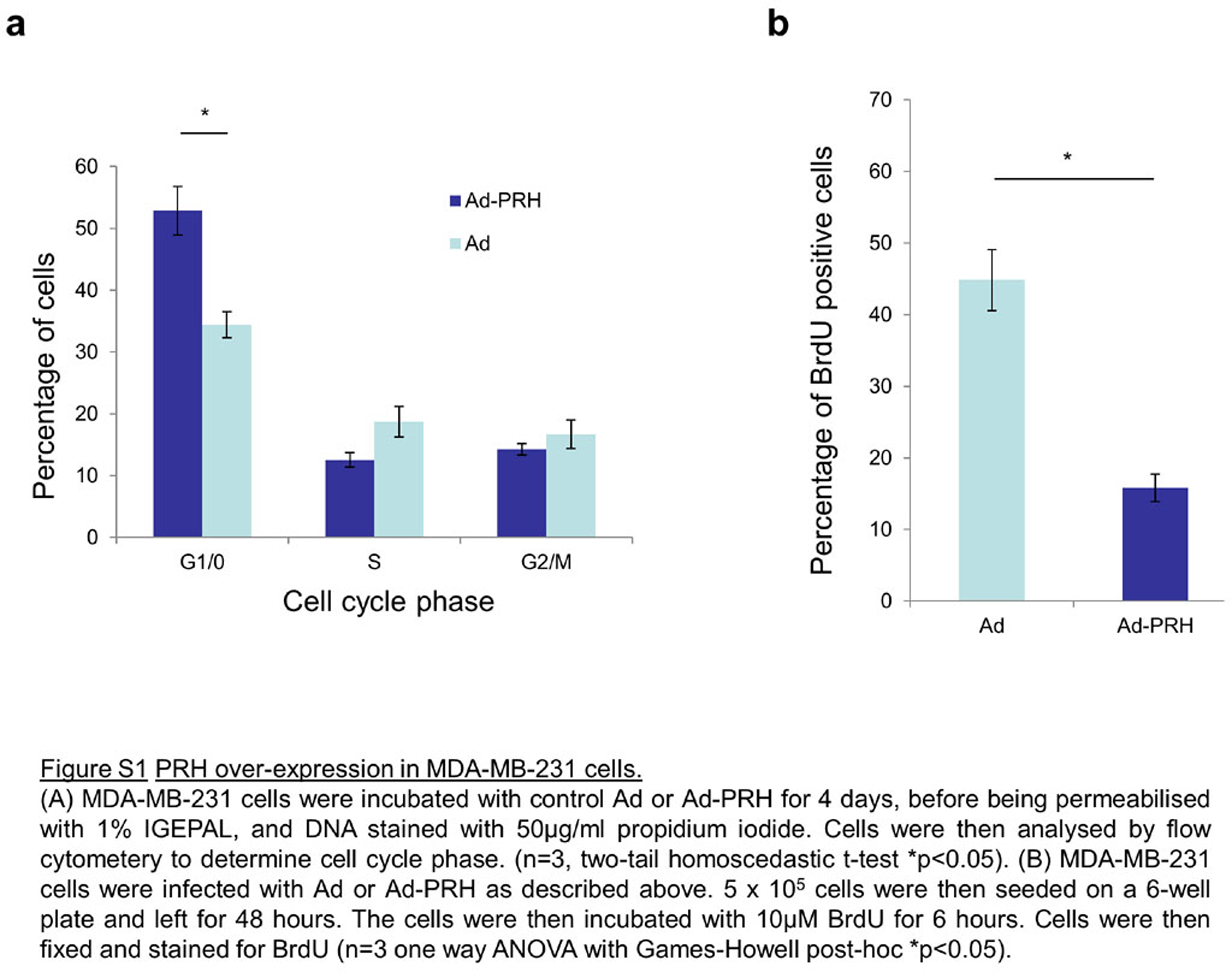

Supplement: Supplementary Figure S1 [file oncsis201742x1.tif]

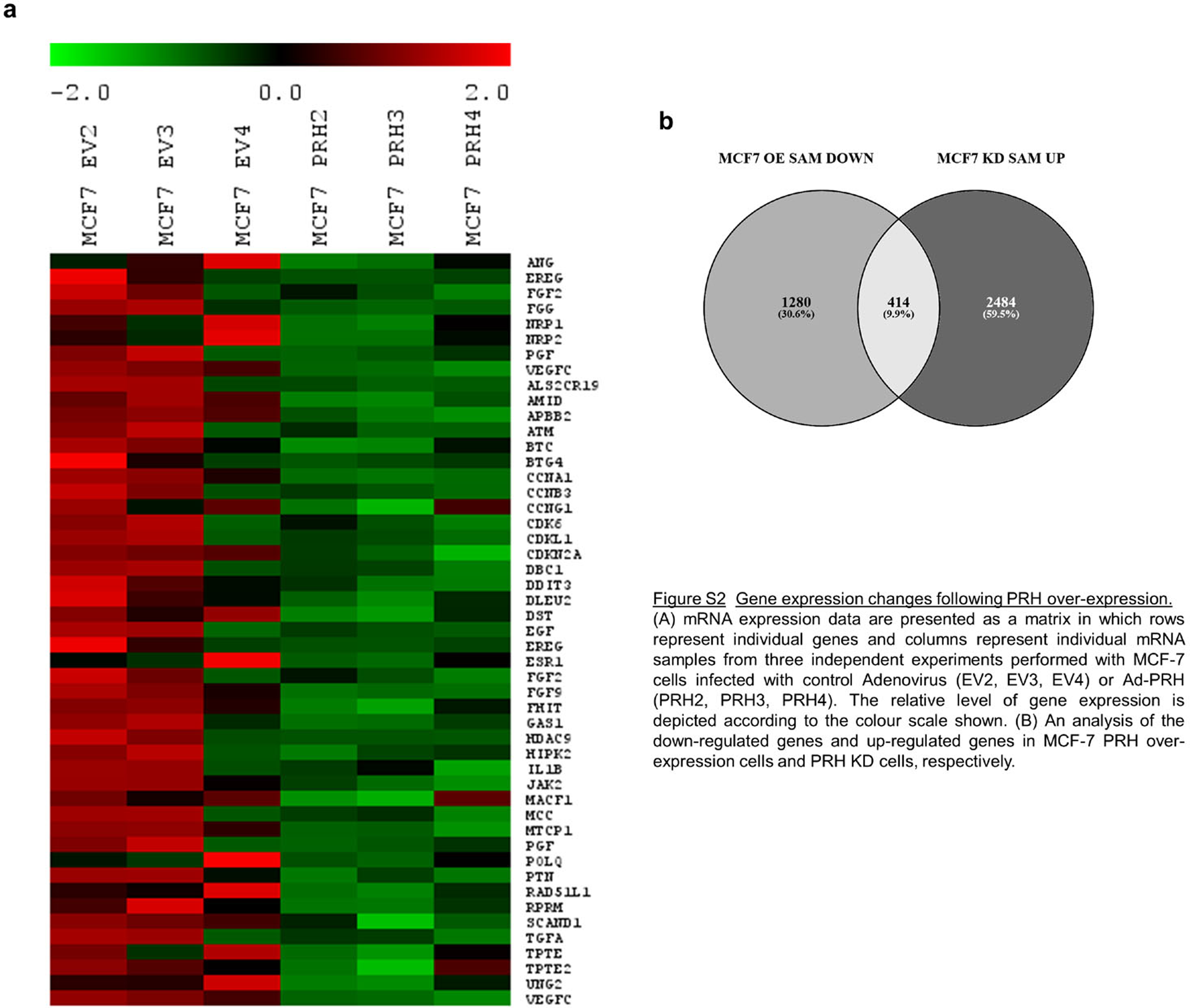

Supplement: Supplementary Figure S2 [file oncsis201742x2.tif]

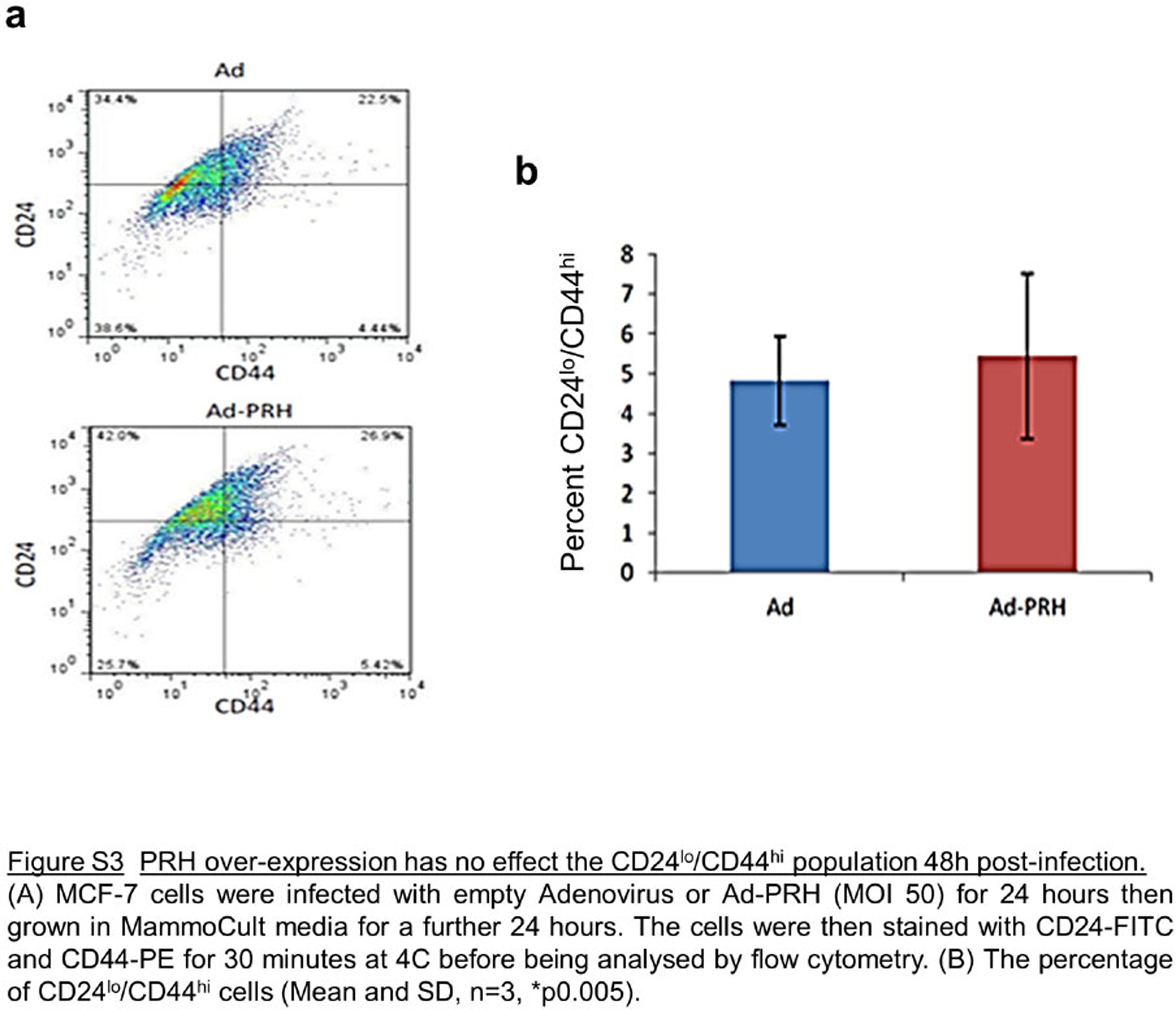

Supplement: Supplementary Figure S3 [file oncsis201742x3.tif]

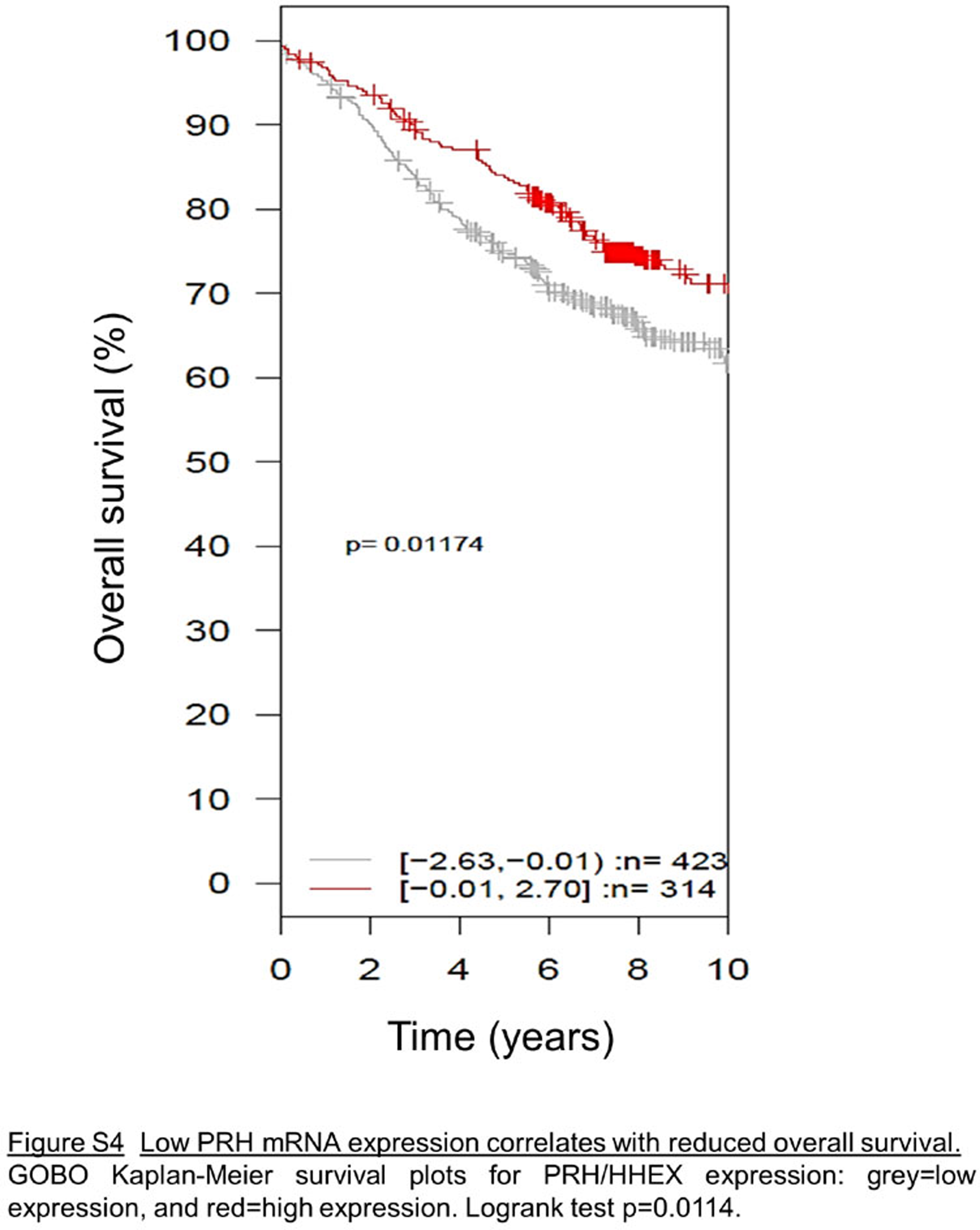

Supplement: Supplementary Figure S4 [file oncsis201742x4.tif]

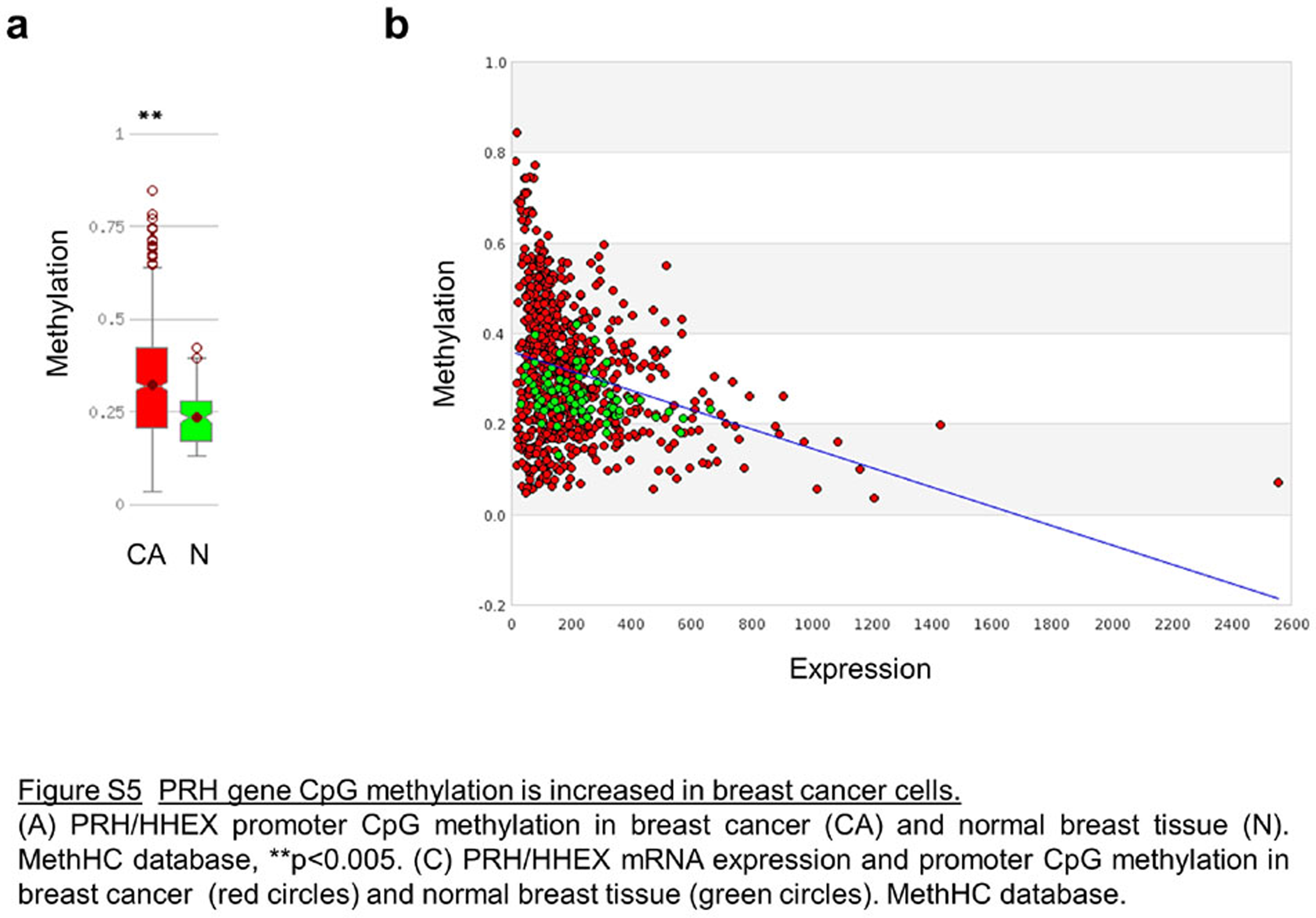

Supplement: Supplementary Figure S5 [file oncsis201742x5.tif]
