# Supplementary material for: Proline-Rich Homeodomain protein (PRH/HHEX) is a suppressor of breast tumour growth
Source: Oncogenesis. 2017 Jun 12;6(6):e346–. doi: 10.1038/oncsis.2017.42 (PMC5519192; doi:10.1038/oncsis.2017.42)
Supplement: Supplementary Table S1 [file oncsis201742x6.docx]

|  | **Forward (5’-3’)** | **Reverse (5’-3’)** |
| --- | --- | --- |
| CCND2 | GGACATCCAACCCTACATGC | CGCACTTCTGTTCCTCACAG |
| CDH1 | GTAACGACGTTGCACCAACC | AGCCAGCTTGAAGCTGAT |
| NRP1 | TATTCCCAGAAGTCTGCCC | TGTCATCCACAGCAATCCCA |
| ENG | GCCGTGCTGGGCATCACCTT | CGCTTGCTGGGGGAACCTGG |
| GAPDH | TGATGACATCAAGAAGGTGGTGAAG | TCCTTGGAGGCCATGTGGGCCAT |
| VEGFC | ATGTGTGTCCGTCTACAGATGT | GGAAGTGTGATTGGCAAAACTGA |
| VEGFA | ATCAGCGCAGCTACTGCCATCC | TCTCCTATGTGCTGGCCTTGGTG |

**Supplemental Tables**

**Table S1.**
